# Association of serum calcium levels with infarct volume and stroke scores in acute ischemic stroke patients: An observational study

**DOI:** 10.22088/cjim.13.4.779

**Published:** 2022

**Authors:** Ramya R Nayak, Srikanth Narayanaswamy

**Affiliations:** 1 Department of General Medicine, Ramaiah Medical College and Hospital, Bengaluru 560 054, Karnataka, India

**Keywords:** Acute stroke, Serum calcium, NIHSS score, Infarct size, Prognosis

## Abstract

**Background:**

Stroke vastly contributes to death and disability worldwide. Acute ischemic stroke (AIS) is caused by a reduction in supply of blood to the brain. Accumulation of unnecessary intracellular serum calcium in AIS induces the cytotoxic actions that activates enzymes involved in cell death. The present investigation assessed the relationship of total serum calcium level (at admission) and initial diffusion weighted imaging (DWI) infarct volume and correlated with National Institute of Health Stroke Scale (NIHSS) scores.

**Methods:**

A hospital-based observational study was conducted on 74 consecutive patients identified with AIS fulfilling the inclusion criteria. NIHSS scores and serum ionized calcium were calculated in every patient and compared with DWI infarct volume for assessing correlation between these three. Statistical software R Version 4.0.2 and Microsoft Excel were used for statistical analysis.

**Results:**

Out of the 74 patients, most of them were in age group of 50-69 years, with a male preponderance (68.9%). A significant association was noticed between diabetes and dyslipidemia with age (P=0.01499). A strong negative correlation was observed between NIHSS scores (at admission & discharge) with ionized calcium, while a strong positive correlation was noticed between stroke scores with infarct volume. A statistically significant negative correlation was recorded between serum calcium (on admission) and infarct size (r=-0.851755, P=0.0001). The mean of NIHSS scores on admission (8.24±5.19) has been remarkably higher when compared with NIHSS scores at discharge (5.25±3.89).

**Conclusion:**

In patients with AIS examined within 6-24 hours of symptoms onset, serum ionized calcium and volume of infarct on DWI showed inverse association. Serum calcium serves as a marker of severity and acts as prognostic factor in AIS.

Stroke vastly contributes to death and disability worldwide (1). Ischemic stroke is triggered by a reduction in blood supply to the brain. Presently, India has high number of new stroke cases than western countries. The very common cause for ischemic stroke in India is large vessel intracranial atherosclerosis (2). The common risk factors such as diabetes, high blood pressure, elevated lipid profile and smoking are relatively predominant and inadequately managed due to poor public consciousness and insufficient infrastructure. In acute ischemic stroke (AIS), increased intracellular serum calcium induces the cytotoxic actions that causes the hyper-activation of several deleterious enzymes and signaling processes that impair neuronal function or lead to cell death (3). Literature shows that low extracellular calcium levels inconsistently boosts this accumulation of intracellular serum calcium that could potentiate cell death (4). 

Yet, the result of serum calcium levels on serum calcium level–dependent excitotoxic pathways in acute cerebral ischemia is not clear. However, the rising research data advise that increased levels of serum calcium at admission are linked with well clinical recovering after AIS (5).

Infarct size is a key determinant of clinical outcome from stroke. Infarct volume, as assessed on either computerized tomography (CT)/magnetic resonance imaging (MRI), has been documented to only moderately correlation with clinical outcomes (6). Analyses of multiple large data sets have revealed that age group and stroke severity, recorded based on the National Institutes of Health Stroke Scale (NIHSS), have significant impact on post-stroke outcomes (7). NIHSS score has helped determine stroke severity, predicted clinical outcome and helps to determine appropriate treatment. In this investigation, we evaluated the relationship between total serum calcium level on admission and initial diffusion weighted imaging (DWI) infarct volume as recorded by MRI in participants with AIS, then, correlated with stroke-related neurological deficit as recorded by NIHSS score.

## Methods

**Study design: **This study was performed on patients hospitalized during October 2017- September 2019 (2 years) at Ramaiah Medical College, Bangalore, India. The investigation was conducted with the approval of ethics committee ((Reference number: SS-1/EC/022/2017) of the institute. A written informed consent taken in English and local language. Patients diagnosed with AIS were included. Patients identified with hemorrhagic stroke, lacunar infarct, temporary ischemic attack, recurring ischemic stroke, imaging >24 hours, those undergoing thrombolysis, malignancy, renal failure, other conditions altering serum calcium levels and patients on calcium supplements ischemic stroke (presented within 6-24 hours of beginning of symptoms) were excluded. The sample size was calculated at 95% confidence level (Z∝/2 is 1.96 and Zβ value for 85% power is 1.0364) to be 67 participants. 

**Code of ethics: **The current study adhered to the code of as prescribed by the international standards (Declaration of Helsinki), local government and the University policies for conducting research. All the principles under code of ethics were met. The study was commenced only after clearance from the board of ethical clearance, Ramaiah Medical College, Bangalore, India. (Reference number: SS-1/EC/022/2017).

**Data collection: **A thorough clinical history and physical examination followed by NIHSS scoring for assessment of neurological status was conducted for patients satisfying the inclusion criteria. NIHSS scores and serum calcium levels were recorded on admission following standard procedures (5, 9). Stroke severity was estimated on admission and discharge. Based on of NIHSS scores, stroke severity was recorded as no stroke (0), minor stroke (1-4), moderate stroke (5-15) and acute stroke (15-41).

A 1.5-Tesla MRI (Magnetom Avento, Siemens Medical Systems, Erlangen, Germany) scanning was done within 24 hours of symptom onset.10 The procedure included diffusion weighted imaging (DWI) and fluid-attenuated inversion recovery imaging (FLAIR). Briefly, DWI has been recorded by radiologist using two stages of diffusion sensitization (b value, 0 & 1000 s/mm^2^) with the variables: 5mm section thickness, no gap and 17-20 sections. DWI infarct volumes were measured following Ellipsoid method.11 Infarct volume = ABC/2

Where, A is the longest dimension in X-axis, B is the longest perpendicular dimension to X-axis and C is the total length in Z-dimension.

**Statistical analysis:** Statistical analyses were performed by the statistical software R version 4.0.2 and MS Excel. Categorical variables were represented by frequency tables and continuous variables were represented in mean ± SD/median (interquartile range) form. Paired t-test/Wilcoxon’s test has been employed to compare mean/ distribution over time points and Mann-Whitney/Kruskal-Wallis test has been employed for comparison of distributions. A p-value (≤0.05) indicated statistical significance. 

## Results

A total of 74 patients with AIS formed the study sample. Table 1 shows the dispersal of patients based on different variables. Out of 74 51 (68.92%) patients, were males and remaining 23 (31.08%) were females. Patients with age of 60-69 years were major in number with 31.08% (23) followed by age 50-59 years with 29.73% (22). Among the study participants, 58.11% (43) patients were diabetics followed by hypertension with 58.11% (43). Among them, 31.08% (23) were using tobacco and 24.32% (18) were alcoholics. When compared with the comorbid conditions with different age sets (table 2), diabetes mellitus and hypertension were found the most common. 

**Table 1.** Descriptive summary of categorical variables

**Variables**		**Number of subjects (%)**
Age (in years)	40-49	11 (14.86%)
	50-59	22 (29.73%)
	60-69	23 (31.08%)
	70-79	12 (16.22%)
	≥ 80	6 (8.11%)
Gender	Male	51 (68.92%)
	Female	23 (31.08%)
Diabetes mellitus	Yes	43 (58.11%)
	No	31 (41.89%)
Hypertension	Yes	43 (58.11%)
	No	31 (41.89%)
Dyslipidemia	Yes	16 (21.62%)
	No	58 (78.38%)
IHD	Yes	7 (9.46%)
	No	67 (90.54%)
Others	Yes	2 (2.7%)
	No	72 (97.3%)
Smoking	Yes	23 (31.08%)
	No	51 (68.92%)
Alcoholic	Yes	18 (24.32%)
	No	56 (75.68%)

IHD, ischemic heart disease

By chi-square test, a significant association was noticed between diabetes and dyslipidemia with age (P=0.01499). But no remarkable correlation between hypertension and IHD with age was observed. Table 3 compares the comorbid conditions over gender, significant variance was noticed in the dispersal of diabetes over gender. Among the diabetics, males were higher in number (34, 79.07%) than females (9, 20.93%). Whereas in hypertensives, 29 (67.44%) were males and 14 (32.56%) were females.

**Figure 1:**
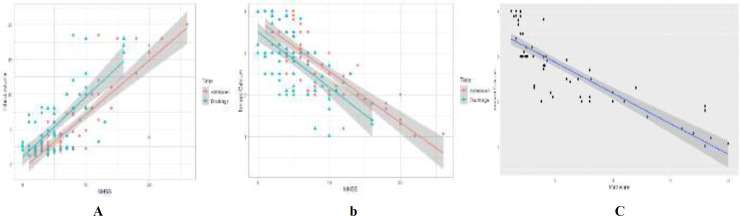
Correlation of (a) NIHSS with ionized calcium on admission and discharge, (b) NIHSS with infarct volume at admission and discharge, and (c) infarct volume and ionized calcium

Different clinical parameters viz., ionized calcium, infarct volume, NIHSS score (on admission & discharge) have been compared with gender and different age group. Significant differences have not been noticed among the parameters tested over age (by ANOVA) and gender (by two sample t- Table 4 describes the association between NIHSS scores at two-time points over ionized calcium and infarct volume. By Pearson’s correlation test, a strong negative correlation observed between NIHSS (on admission & discharge) with ionized calcium (figure 1a). While a strong positive correlation noticed between NIHSS (on admission & discharge) with infarct volume (figure 1b). In comparison of ionized calcium and infarct volume, a strong negative correlation observed between ionized calcium and infarct volume (r= -0.851755, P= <0.0001) (figure 1c).

On admission, 50 (67.6%) patients had NIHSS score <10 and 2 (2.7%) patients had score >20; while at discharge, 61 (82.4%) patients had score of <10 and no one had score of >20. In hospital, death occurred in 2 patients. Table 5 represents the mean of NIHSS scores at the admission and discharge. By one-tailed paired t-test, it was recorded that the mean of NIHSS scores on admission (8.24±5.19) has been remarkably higher when compared with NIHSS scores at discharge (5.25±3.89). Majority of patients showed improvement in neurological problems by discharge that was measured by NIHSS scores at the discharge and was compared to the score on admission. 

**Table 2:** Association of comorbid conditions of patients in relation to age distribution

**Comorbid conditions**		**Age (in years)**					**P-value**
		**40-49**	**50-59**	**60-69**	**70-79**	**≥ 80**	
Diabetes mellitus	Yes	3 (6.98%)	10(23.26%)	16(37.21%)	11(25.58%)	3 (6.98%)	0.01499*MC
	No	8 (25.81%)	12(38.71%)	7 (22.58%)	1 (3.23%)	3 (9.68%)	
Hypertension	Yes	4 (9.3%)	11(25.58%)	16(37.21%)	9 (20.93%)	3 (6.98%)	0.2384MC
	No	7 (22.58%)	11(35.48%)	7 (22.58%)	3 (9.68%)	3 (9.68%)	
Dyslipidemia	Yes	3 (18.75%)	9 (56.25%)	0 (0%)	3 (18.75%)	1 (6.25%)	0.01499*MC
	No	8 (13.79%)	13(22.41%)	23(39.66%)	9 (15.52%)	5 (8.62%)	
IHD	Yes	0 (0%)	2 (28.57%)	2 (28.57%)	2 (28.57%)	1(14.29%)	0.7351MC
	No	11(16.42%)	20(29.85%)	21(31.34%)	10(14.93%)	5 (7.46%)	

IHD, ischemic heart disease; *, statistically significant; MC, Monte-Carlo’s simulation

**Table 3:** Association of comorbid conditions of patients in relation to gender

**Comorbid conditions**		**Gender**		**P-value**
		**Male**	**Female**	
Diabetes mellitus	Yes	34 (79.07%)	9 (20.93%)	0.02628*
	No	17 (54.84%)	14 (45.16%)	
Hypertension	Yes	29 (67.44%)	14 (32.56%)	0.7464
	No	22 (70.97%)	9 (29.03%)	
Dyslipidemia	Yes	10 (62.5%)	6 (37.5%)	0.5502MC
	No	41 (70.69%)	17 (29.31%)	
IHD	Yes	5 (71.43%)	2 (28.57%)	1MC
	No	46 (68.66%)	21 (31.34%)	

IHD, ischemic heart disease; *, statistically significant; MC, Monte-Carlo’s simulation

**Table 4:** Correlation of NIHSS with ionized calcium and infarct volume on admission and discharge of patients

**Variable**		**Correlation with ionized calcium**	**P-value**	**Correlation with infarct volume**	**P-value**
NIHSS	At admission	r = -0.807576	<0.00001*	r = 0.8212408	<0.00001*
	At discharge	r = -0.6710315	<0.00001*	r = 0.716707	<0.00001*

NIHSS, National Institute of Health Stroke Scale; *, statistically significant

**Table 5:** Comparison of NIHSS scores at admission and discharge

**Variable**		**Mean± SD**	**P-value**
NIHSS	On admission	8.24±5.19	<0.00001*
	On discharge	5.25±3.89	

NIHSS, National Institute of Health Stroke Scale; *, statistically significant

## Discussion

The immense burden of AIS exhibiting the need to develop precise estimates of a stroke survivor’s prognosis remains an important goal (12). In recent days, many researchers have been focusing on investigating the connection between serum calcium and AIS. This study intends to correlate that high levels of serum calcium at admission will be independently linked with minor infarct sizes in MRI. The disease incidence was dominant in patients with age 60-69 years followed by 50-59 years; 68.92% were males and remaining 31.08% were females. Aging and sex are critical factors in pathology of AIS (13). Aging is the strongest non-modifiable risk factor for stroke incidences with doubling for every 10 years post the age of 55 and, almost three-quarters of all stroke incidences occur in persons aged ≥65 years (14). In epidemiology of AIS, sex differences are age-dependent since the sex influences on stroke risk and outcome changes across the lifespan. In early adulthood, AIS incidences are higher in men with poor functional results than women; whereas, AIS incidences commence to increase at the middle age in women, linked with menopause and deprived female sex hormones (13).

Diabetes mellitus (58.11%) and hypertension (58.11%) were found to be the major comorbid conditions. By Chi-square test, an important association observed among diabetes and dyslipidemia with age, but the similar association has not been observed between hypertension and IHD with age. A higher occurrence of diabetes and elevated blood pressure stimulates the AIS incidence in the aged population (13). Diabetes, hypertension, smoking and dyslipidemia have been observed as major changeable risk factors for AIS. In addition, diabetes and high blood pressure are coexistent modifiable risk factors for stroke (15). In this investigation, the percentage incidences of diabetes and hypertension were almost similar, these results are in agreement with the previous statement. Comparison of clinical parameters namely, ionized calcium, infarct volume, NIHSS (on admission and discharge) showed the relationship among each other. Evidence from the previous reports suggests that the serum calcium at admission & infarct volume, and serum calcium & NIHSS score at the admission have been correlated with each other (5, 16, 17-19). These results are concurring with the previous findings described in the Indian population. 

In intracerebral hemorrhage, the decreased level of serum calcium on admission indicated poor prognosis (90-day death or major disability) and the probable mechanism involved was calcium-induced coagulation function abnormality (20) Lower levels of serum calcium may be linked with more critical clinical findings at onset in patients severe stroke and reflects the severity of ischemic injury (21). An investigation in 2019 by Liu et al. reported that when the blood calcium level has been too low or too high, hematoma volume and severity of stroke may increase in severe cerebral hemorrhage patients, and it has been correlated with long-term survival (22). 

Hence, the serum calcium can be regarded as an indicator for AIS, however, it also can play a newfangled role as an extrapolative marker in prognosis of AIS. This study has few potential limitations. The study has comparatively low sample size. And, the impact of initial and delayed calcium levels on clinical success after AIS has not been reported.

In conclusion a remarkable negative correlation has been found between (i) serum calcium on admission and infarct volume, and (ii) serum calcium and NIHSS on admission. This finding implies that decreased ionized calcium on admission is linked with increased infarct size and increased NIHSS on admission. Serum calcium could be a potential marker to estimate the seriousness of neurological deficit in AIS. Further investigations with a larger sample size and direct measurement of calcium levels at specified time intervals are necessary to comprehend the pathophysiological mechanism of AIS.
